# A Clinicopathological Study of Marginal Gingival Leukoplakia

**DOI:** 10.1111/jop.70052

**Published:** 2025-09-04

**Authors:** Daniel Lobato Ferreira Ferraz, Caique Mariano Pedroso, Hélen Kaline Farias Bezerra, Alan Roger dos Santos‐Silva, Pablo Agustin Vargas, Marcio Ajudarte Lopes

**Affiliations:** ^1^ Oral Diagnosis Department Piracicaba Dental School, University of Campinas (UNICAMP) Piracicaba Brazil

**Keywords:** leukoplakia, marginal gingiva, proliferative verrucous leukoplakia, ring around the collar, squamous cell carcinoma, treatment outcome

## Abstract

**Background:**

Marginal gingival leukoplakias are relatively uncommon and rarely discussed in the scientific literature. Studies suggest they are distinct from other leukoplakias due to aggressive behavior and a strong association with proliferative verrucous leukoplakia. This study aimed to evaluate the clinicopathological characteristics of patients diagnosed with marginal gingival lesions.

**Methods:**

This retrospective study analyzed 32 patients diagnosed with marginal gingival leukoplakia. Clinicopathological data were extracted. K‐means clustering and principal component analyses identified subgroups within the dataset. Histopathological findings were assessed by two oral pathologists using World Health Organization criteria for grading oral epithelial dysplasia.

**Results:**

The lesions predominantly affected older individuals (mean age: 60.4 years), 16 men and 16 women, and exhibited multifocality in 75% of cases. Proliferative verrucous leukoplakia was diagnosed in 24 patients (75%), and most lesions were homogeneous (84.4%). Thirteen patients were treated (three scalpel, eight laser, two both), while 19 underwent “wait and see.” Over a 95.4‐month average follow‐up, 11 recurrences (73.3%) were noted: four after scalpel (80%) and seven after laser excision (70%). Malignant transformation occurred in three cases. The most common histopathological feature was hyperkeratosis (*n* = 24), and eight cases showed mild, one moderate, and two severe epithelial dysplasia. Cluster analysis revealed five subgroups.

**Conclusion:**

Marginal gingival leukoplakias demonstrate significant heterogeneity but seem to be strongly associated with proliferative verrucous leukoplakia. Recurrence is a common outcome, and laser excision might be a better option for lesion control. Close monitoring remains essential for early intervention and improved outcomes.

## 1 Introduction

Oral leukoplakia (OL) is the most prevalent oral potentially malignant disorder (OPMD) worldwide [1]. A subtype of this group, proliferative verrucous leukoplakia (PVL) is less common but is generally more aggressive and usually affects a group of patients with different characteristics from those affected by conventional OL, such as older women who are not tobacco smokers [2, 3]. Most PVL lesions occur on the gingiva, tongue, and buccal mucosa, with a varying verrucous aspect that can usually become thicker and more exuberant over time [2, 3, 4].

In this scenario, a pattern of leukoplakia has caught attention due to its peculiar clinical presentation and more aggressive behavior: the marginal gingival leukoplakia (MGL). This group of lesions has been associated as an early manifestation of PVL by some authors, since it has been observed that in many cases the white patches first appear on the marginal or attached gingiva, affecting other sites over time [5, 6, 7]. Although PVL is recognized for its multifocal progression and resistance to treatment, the nature of localized leukoplakias on the marginal gingiva has not yet been fully understood, with studies or case reports on this topic being scarce, raising questions about their true significance within the spectrum of OPMDs.

One of the first studies addressing the clinicopathological features of gingival leukoplakias was conducted by Fettig et al. [8], revealing that PVL lesions found on the marginal or attached gingiva are typically solitary, presenting as verrucous white plaques that exhibit a high tendency for recurrence. The authors suggested that these lesions appear to be a subtype of PVL and are characterized by an unpredictable clinical progression and a significant risk of malignant transformation (MT) into verrucous or squamous cell carcinoma [8]. Furthermore, other scientific literature in the last years, including an editorial by Lingen [5], has continued to explore this topic, introducing the term “ring around the collar,” which was coined by Professor Donald Cohen to describe the MGLs.

An observational study conducted by Upadhyaya et al. [6] also analyzed the clinical and histopathological aspects of these lesions. The findings indicated that, although MGLs may initially resemble small benign lesions, they exhibit distinct clinical behavior compared to other conventional OL, characterized by higher recurrence rates following surgical excision and substantial MT rates [6]. Furthermore, the study reinforced the theory that they appear to be an early sign of PVL and highlighted the tendency of professionals to often misdiagnose them as benign conditions, such as frictional hyperkeratosis, which can negatively impact the prognosis of such cases [6].

Regarding the MT of these lesions, some studies revealed a higher rate for gingival PVL lesions than in other sites, reaching up to 73% of cases, and this outcome does not seem to be related to any of the risk factors already recognized for oral squamous cell carcinoma [9]. However, most of the few published studies focusing solely on MGL until now lacked sufficient follow‐up time to analyze this type of outcome or did not include a significant group of patients with sufficiently reported characteristics to make clinicopathological correlations.

Therefore, our study aimed to assess the main clinicopathological characteristics of MGL in a group of patients in order to improve our knowledge about its nature, association with PVL, treatment, and possible features associated with its recurrence and MT.

## 2 Materials and Methods

### 2.1 Study Population

This study was conducted after approval by the Research Ethics Committee of the Piracicaba Dental School, University of Campinas (ethical committee approval no. 69909323.5.0000.5418), and all patients included in the research provided informed consent for their participation. Data were collected from patients diagnosed with OL between 2000 and 2024 at the Oral Medicine Clinic of the same University. Their data were accessed retrospectively through an electronic medical records database. From this sample, the inclusion criteria included cases diagnosed as OL involving the marginal gingiva, with their clinical images and complete data, as well as their histopathological analysis, if available. A minimum clinical follow‐up period of 3 months was required for inclusion in the present study. Exclusion criteria consisted of (1) cases of OL with no involvement of marginal gingiva, (2) cases with no clinical images and insufficient data for analysis.

### 2.2 Clinical Data Extraction

The extracted data, when available, included characteristics of the sample of patients, such as gender, age, habits (tobacco smoking, alcohol consumption, trauma), systemic alterations, clinical appearance, size, location, and time of evolution of the lesions, interventions, and outcomes (recurrence and MT). All the data were tabulated in Microsoft Excel. The clinical images were also accessed to confirm the involvement of marginal gingiva.

### 2.3 Histopathological Data Extraction

The included cases with available histopathological analysis had their slides stained in hematoxylin and eosin (H&E) retrieved, and microscopically analyzed by two calibrated oral pathologists. The following data were collected: presence of hyperkeratosis, epithelial hyperplasia, and subepithelial inflammatory infiltrate. Oral epithelial dysplasia, when present, was also graded according to the cellular and architectural criteria of the World Health Organization [2]. The data were tabulated in Microsoft Excel.

### 2.4 Statistical Analysis

All analyses were conducted using R software with a significance level set at 0.05 to determine statistical significance. A descriptive analysis was performed to summarize the clinicopathological characteristics of the patients. The Kappa statistic was used to assess interobserver agreement for grading the epithelial dysplasia in the cases, and scores were interpreted based on the criteria proposed by Landis and Koch, where values of 0–0.2 indicate slight agreement, 0.2–0.4 fair agreement, 0.4–0.6 moderate agreement, 0.6–0.8 substantial agreement, and 0.8–1.0 almost perfect agreement [10]. Fisher's exact test was employed to compare the MGLs associated with PVL and those not associated with PVL. K‐means clustering analysis was performed to identify groups in the dataset related to leukoplakia characteristics and treatments. This unsupervised machine learning technique divides data into separate clusters according to their similarities, facilitating the recognition of patterns and the classification of subgroups within the dataset. Additionally, a principal component analysis (PCA) was conducted to reduce the dimensionality of the dataset while capturing most of the variance. The Kruskal–Wallis test was used to assess differences among clusters, followed by Dunn's post hoc test for significant variables to determine specific group differences.

## 3 Results

### 3.1 Screening and Demographic Characteristics

During the screening phase, 258 patients diagnosed with OL between 2000 and 2024 were retrieved from the electronic medical records database at the Oral Medicine Clinic of the Piracicaba Dental School, University of Campinas. After applying the inclusion and exclusion criteria, the final sample totaled 32 patients diagnosed with MGLs, 16 men and 16 women, with a mean age of 60.4 (29–87) years. Regarding risk habits for OL progression, only four patients were current tobacco smokers and six had a tobacco smoking history. Six patients were alcohol consumers. One patient reported vigorous tooth brushing with a hard toothbrush for a long time. Concerning systemic alterations, 11 patients had hypertension, three had diabetes and hypothyroidism, respectively. Fifteen patients reported no systemic alterations. Demographic characteristics of the sample are detailed in Table 1.

**TABLE 1:** Clinical and demographic features of patients with MGLs and their association with the diagnosis of PVL.

Features	PVL *N* (%)	Non‐PVL *N* (%)	Total *N*	*p* ^a^
Gender				
Male	13 (54.2)	3 (37.5)	16	0.7
Female	11 (45.8)	5 (62.5)	16	
Age				
Age < 50 years	3 (12.5)	2 (25)	5	0.6
Age > 50 years	21 (87.5)	6 (75)	27	
Habits				
Tobacco smoking	4 (16.7)	0 (0)	4	0.5
Alcohol consumption	4 (16.7)	2 (25)	6	0.6
Clinical aspect				
Homogeneous	19 (79.2)	8 (100)	27	0.3
Nonhomogeneous	5 (20.8)	0 (0)	5	
Lesion size				
> 2 cm	11 (45.8)	1 (12.5)	12	0.3
< 2 cm	13 (54.2)	7 (87.5)	20	
Multifocal	24 (100)	0 (0)	24	**0.0009**
Location (for multifocal lesions)				
Alveolar ridge	3 (12.5)	0 (0)	3	1.0
Buccal mucosa	6 (25)	0 (0)	6	1.0
Tongue	2 (8.3)	0 (0)	2	1.0
Mouth floor	2 (8.3)	0 (0)	2	1.0
Palate	2 (8.3)	0 (0)	2	1.0
Attached gingiva	5 (20.8)	0 (0)	5	1.0

*Note: p* value is statistically significant.

Abbreviations: MGL, marginal gingival leukoplakia; PVL, proliferative verrucous leukoplakia.

^a^ Fisher test (*p* > 0.05).

### 3.2 Clinical and Management Characteristics

**FIGURE 1:**
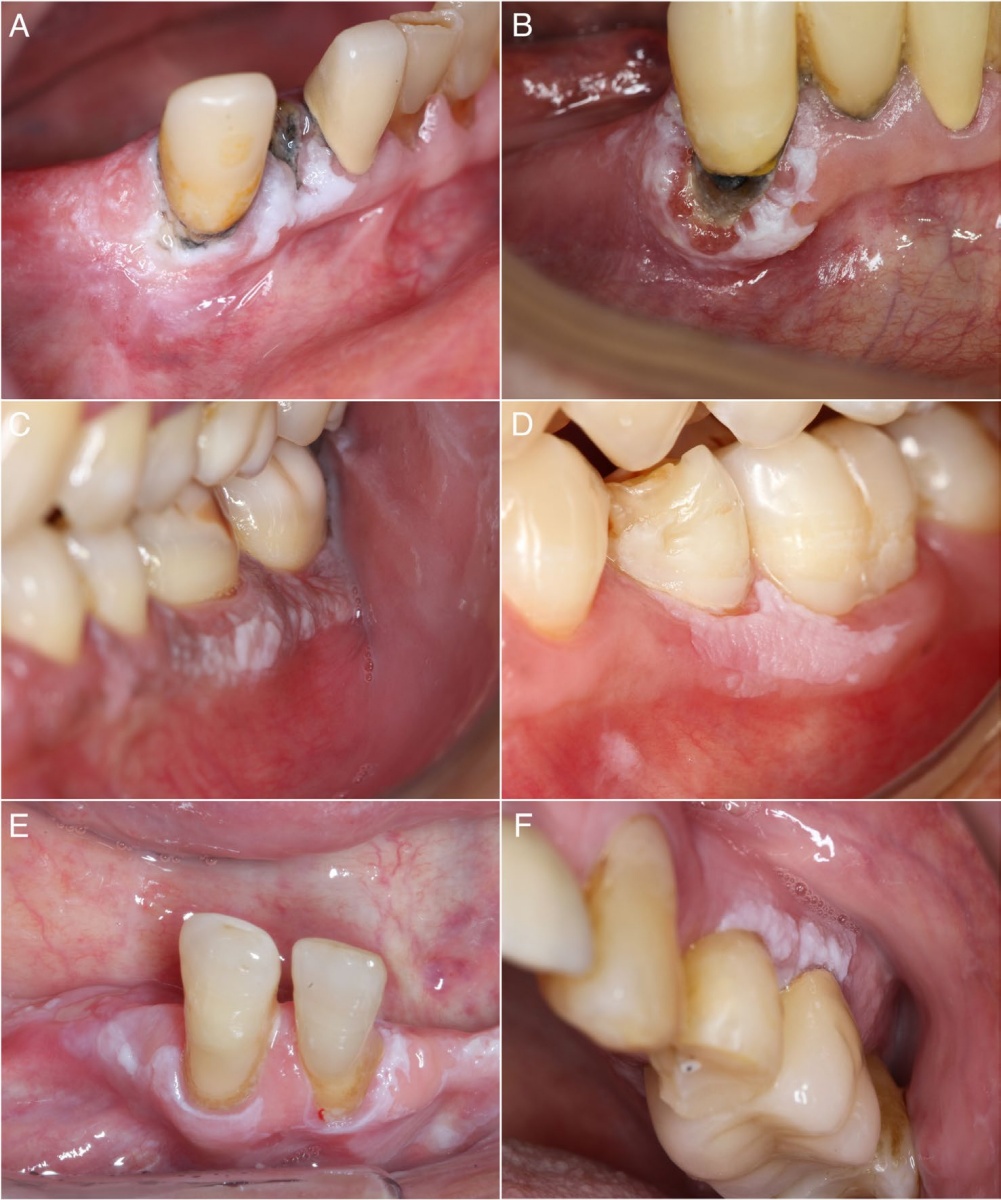
Clinical presentation of marginal gingival leukoplakias. (A) Homogeneous leukoplakia adjacent to the cervical region of lower anterior teeth in a classic “ring around the collar” pattern. (B) Nonhomogeneous leukoplakia encircling the cervical region of lower anterior tooth. (C) Homogeneous leukoplakia extending along the marginal gingiva from the lower premolar to the molar region with thick areas of hyperkeratosis. (D) Homogeneous leukoplakia affecting the lower posterior gingiva with well‐defined borders. (E) Homogeneous leukoplakia encircling the cervical region of lower anterior teeth extending to the adjacent alveolar ridge. (F) Focal homogeneous leukoplakia with thick and verrucous aspect on the upper posterior marginal gingiva.

Regarding the clinical characteristics of the lesions, 24 patients presented with multifocal leukoplakias in sites beyond the marginal gingiva, with the buccal mucosa being the most affected, followed by the attached gingiva, alveolar ridge, tongue, palate, and mouth floor. Most lesions were homogeneous (*n* = 27), with only five classified as nonhomogeneous. The average size of the MGLs was 1.4 cm. Following Villa et al.'s criteria [11], 24 cases (75%) were classified as PVL, with the clinical feature most strongly associated with this diagnosis being the presence of multifocal lesions (*p* = 0.0009). The remaining cases were diagnosed as conventional leukoplakias after differential diagnosis of other white lesions and failure to meet Villa et al.'s criteria. The clinical characteristics of the sample are detailed in Table 1 and Figure 1. Thirteen patients underwent treatment, with three cases being exclusively treated with scalpel excision, eight with laser excision, and two with both modalities at different times. The other patients didn't receive any treatment modality (“wait and see,” *n* = 19). The patients' follow‐up period averaged 95.4 (3–252) months. During this period, 11 recurrences (73.3%) were observed post‐treatment. This outcome was observed in four PVL cases (80%) following scalpel excision, with a mean follow‐up time of 5.3 months, and in seven cases (70%) after laser excision, of which six were PVL lesions, with an average follow‐up time of 8.6 months (Table S1). Three patients had a history of MT of their lesions: two were referred for evaluation of suspicious gingival growths, which were biopsied and confirmed as squamous cell carcinoma. The third case progressed from a gingival erythroleukoplakia after a 9‐month follow‐up (Table S1). These three patients had multifocal lesions, including additional MGLs, and their carcinomas were treated mainly with extensive mandibular resections.

### 3.3 Histopathological Characteristics

**FIGURE 2:**
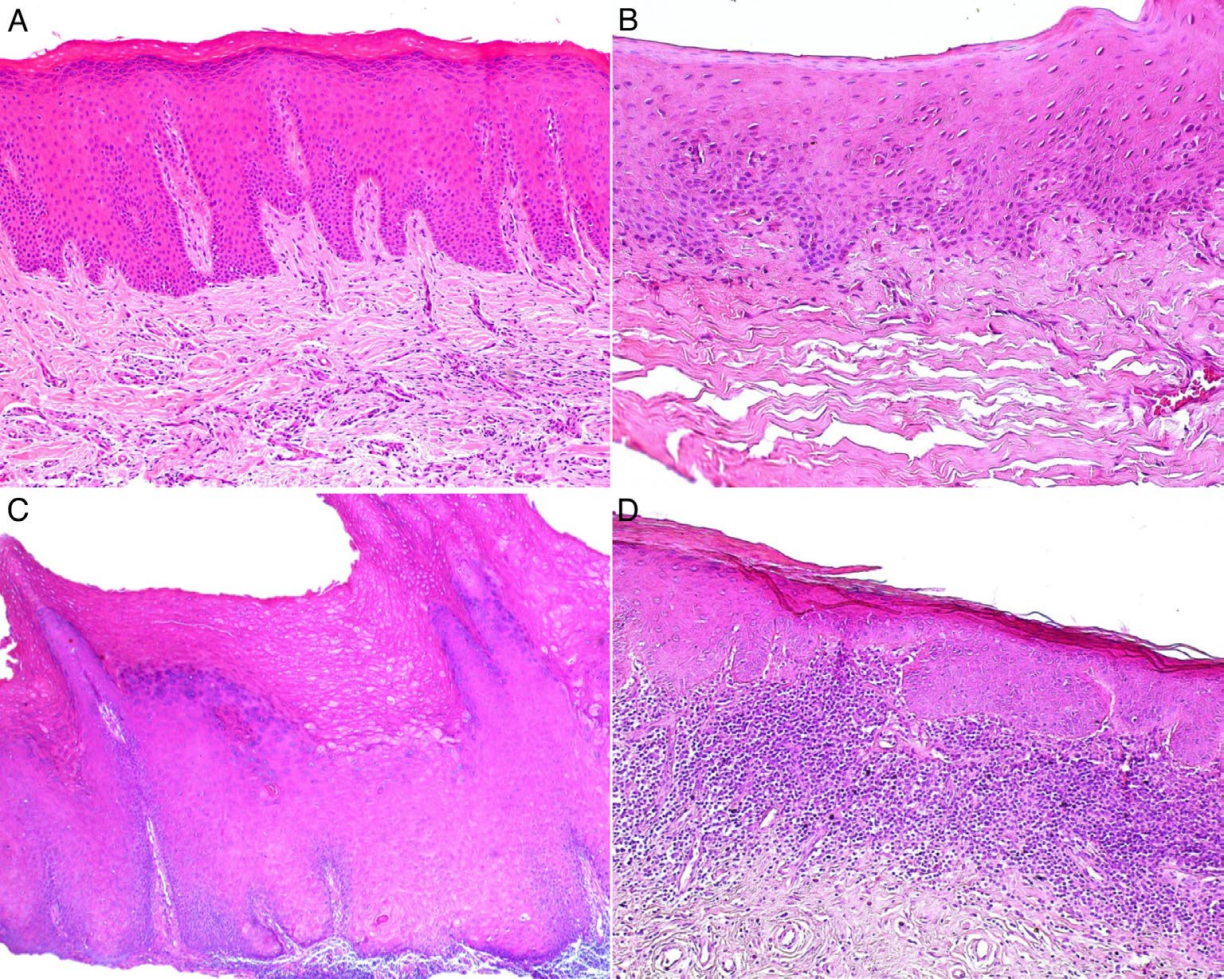
Histopathological features of marginal gingival leukoplakias, H&E. (A) Hyperkeratinized squamous epithelium with areas of acanthosis, without epithelial dysplasia (100×). (B) Parakeratinized squamous epithelium with mild epithelial dysplasia—cellular alterations confined to the lower third (200×). (C) Hyperkeratinized squamous epithelium with acanthosis and verrucous architecture, displaying moderate epithelial dysplasia—cellular and architectural alterations limited to the middle third (50×). (D) Hyperkeratinized squamous epithelium with severe epithelial dysplasia—cellular and architectural alterations extending to the upper third, including drop‐shaped rete ridges; intense subepithelial lymphocytic inflammatory infiltrate (100×).

Of the 32 patients included in the study, histopathological analyses were available for 24. Regarding the most observed histopathological features in the sample, all microscopically analyzed lesions exhibited hyperkeratosis (*n* = 24) (Figure 2A), and 19 cases presented with epithelial hyperplasia (acanthosis) (Figure 2A). The analysis of the Cohen's Kappa coefficient to assess the level of agreement between observers for grading epithelial dysplasia in the lesions yielded a score of 0.8 (*p* = 0.01), indicating a substantial level of agreement. Eight cases demonstrated a subepithelial lymphocytic inflammatory infiltrate (Figure 2D). Concerning cellular atypia, eight lesions were classified as having mild epithelial dysplasia (Figure 2B), one case was classified as moderate epithelial dysplasia (Figure 2C), and two cases were graded as severe (Figure 2D). The distribution of histopathological characteristics is detailed in Table 2.

**TABLE 2:** Histopathological features of MGLs.

Features	*N* (%)
Total	24 (100)
Hyperkeratosis	24 (100)
Epithelial hyperplasia	19 (79.2)
Subepithelial lymphocytic inflammatory infiltrate	8 (33.3)
Mild epithelial dysplasia	8 (33.3)
Moderate epithelial dysplasia	1 (4.2)
Severe epithelial dysplasia	2 (8.3)

Abbreviation: MGL, marginal gingival leukoplakia.

### 3.4 Quantitative Analysis

The K‐means clustering analysis identified five distinct clusters, each characterized by unique demographic, clinical, and treatment‐related attributes. The sizes of the clusters were approximately 3, 8, 6, 6, and 4 individuals, respectively. Cluster 1 was predominantly composed of older females with homogeneous and multifocal lesions, exhibiting a high prevalence of PVL and a preference for laser excision treatment. Cluster 2 showed balanced characteristics between male and female genders, with a higher proportion of non‐PVL homogeneous lesions undergoing “wait and see,” and a notable variability in alcohol consumption. Cluster 3 was characterized by a high proportion of males, with significant tobacco use and homogeneous leukoplakia lesions, predominantly treated with scalpel excision. Cluster 4 was associated with a higher prevalence of laser excision of non‐homogeneous PVL lesions, primarily involving specific areas such as the buccal mucosa and gingiva, and MT. Lastly, Cluster 5 had a predominance of females and older individuals, with a higher presence of multifocal PVL lesions, managed predominantly through laser excision, and a high frequency of outcomes such as recurrence and MT. The clustering results explained 40.6% of the total variance (22.5% by Dim1 and 18.1% by Dim2), indicating that the clusters effectively captured meaningful patterns within the data.

**FIGURE 3:**
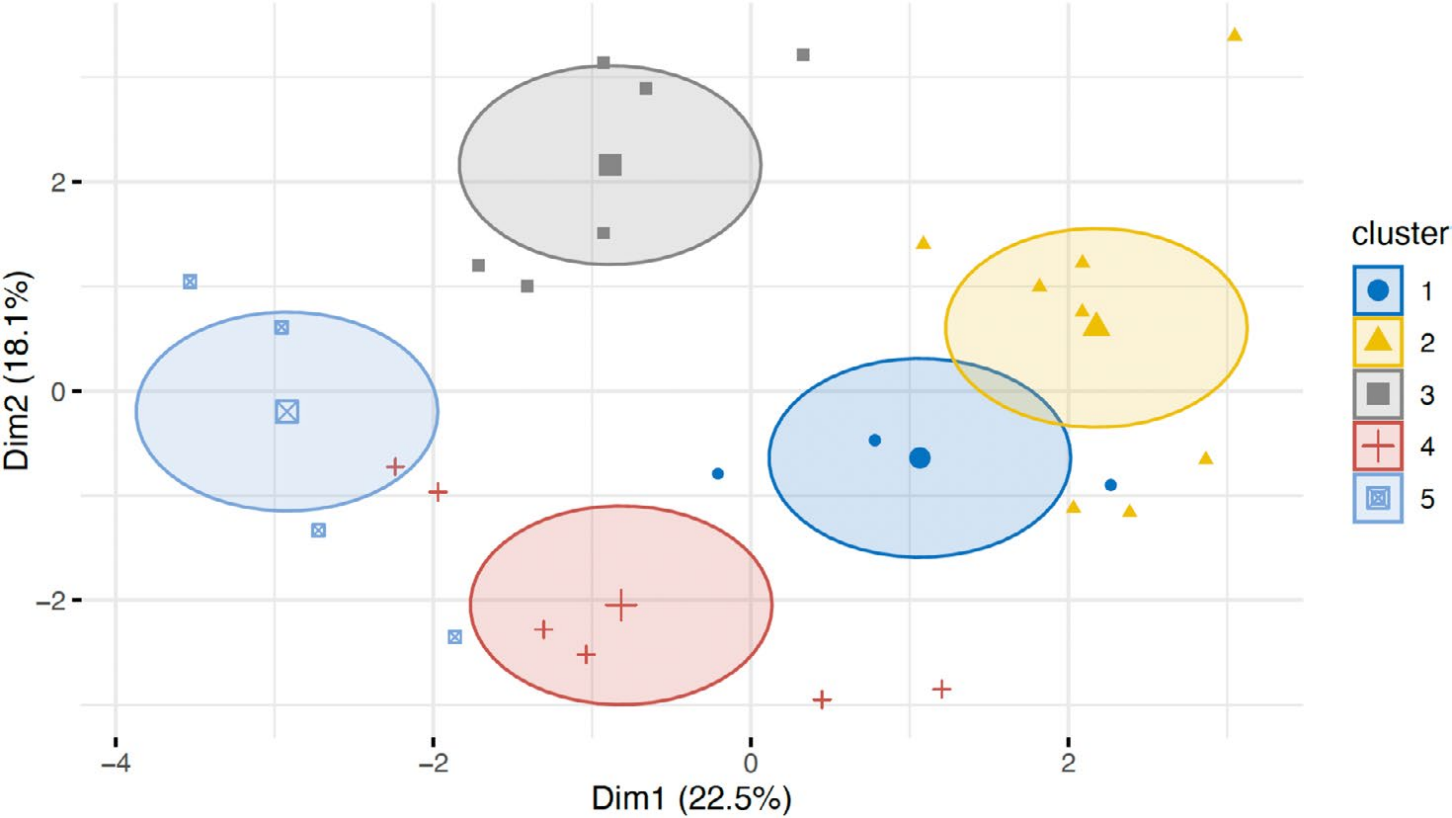
Distribution of the five patient clusters obtained by *k*‐means clustering, displayed on the first two principal components (Dim1 = 22.5% of variance; Dim2 = 18.1% of variance). Each point represents a single patient, with colors and shapes indicating cluster membership. Ellipses represent 95% confidence intervals for each cluster.

**FIGURE 4:**
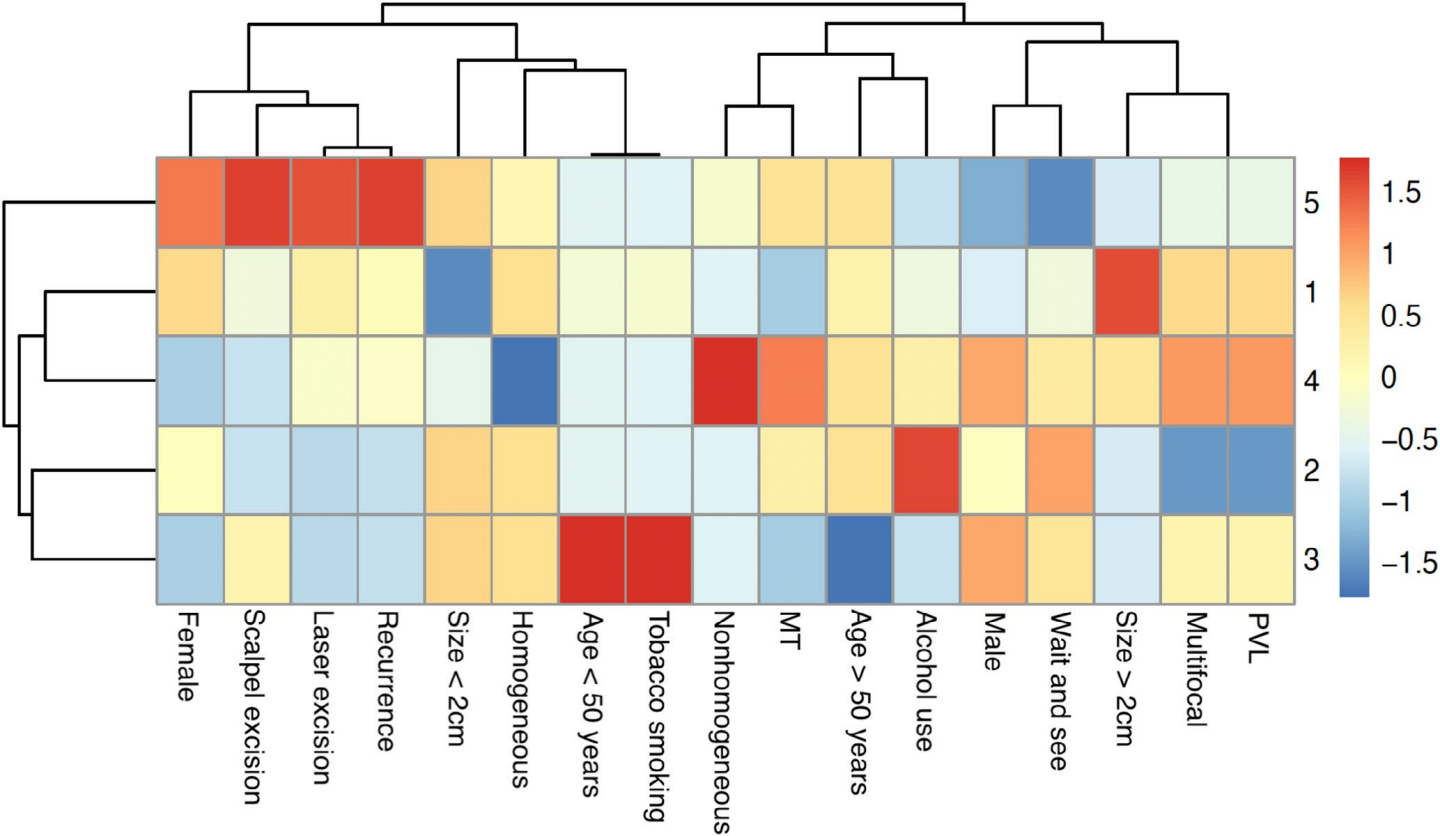
Heatmap showing the relative prevalence of clinical and demographic variables across the five patient clusters. Warmer colors (red) indicate higher prevalence, cooler colors (blue) indicate lower prevalence, and yellow tones represent intermediate values. Variables and clusters are arranged by hierarchical clustering to highlight patterns of association. MT, malignant transformation; PVL, proliferative verrucous leukoplakia.

K‐means clustering analysis also revealed distinct groupings within the dataset, visualized in both a scatter plot (Figure 3) and a heatmap (Figure 4). The scatter plot illustrated a clear separation between clusters along two principal dimensions (Dim1 and Dim2), accounting for 22.5% and 18.1% of the variance, respectively. This separation suggests that the variables form distinct clusters with minimal overlap. The heatmap highlighted associations among variables, such as the clustering of “Age > 50 years,” “MT,” and “Nonhomogeneous” characteristics, while “Homogeneous” and “Wait and see” showed less correlation.

The Kruskal–Wallis test, complemented by post hoc Dunn's results, revealed statistically significant differences across clusters for multiple variables. For “Gender,” a *p*‐value of 0.005 indicated significant differences, particularly between clusters 1 vs. 3 and 1 vs. 5. For “Age,” a *p*‐value < 0.0001 confirmed significant differences between clusters. “Lesion size” also varied significantly (*p* = 0.01), with post hoc analysis showing differences between clusters 1 vs. 4. “Multifocality” demonstrated a *p*‐value of 0.00005, indicating significant variation across clusters. Similarly, treatment modalities such as scalpel excision (*p* = 0.03) and laser excision (*p* = 0.02) showed significant differences. For the variable “Wait and see” a *p*‐value of 0.002 indicated notable differences, particularly between clusters 1 vs. 2 and 2 vs. 3. Finally, “Recurrence” exhibited significant differences (*p* = 0.00002) across clusters, underscoring variability in outcomes.

## 4 Discussion

PVL was first described in the scientific literature over almost 40 years ago by Hansen et al. [12], and since then, numerous studies have sought to elucidate the clinical and histopathological features of this group of lesions. Within the category of OPMDs, PVL stands out as one of the most likely to suffer MT [2, 3, 4]. Some authors have highlighted that many PVL lesions initially manifest on the marginal gingiva, progressing to a “ring around the collar” pattern that often becomes multifocal over time [5, 6]. Gingival leukoplakias, when considered in the context of PVL, have even been suggested as a specific subtype of lesion due to their distinct characteristics [8].

The findings of this study provide significant insights into the clinical and pathological behavior of MGLs, and to the best of the authors' knowledge, this observational research addresses the largest sample of patients published in the English‐language literature. Consistent with previous studies, our results demonstrate that these lesions are predominantly multifocal, often involving other oral sites, and are strongly associated with PVL [5, 6, 7]. This supports the hypothesis that MGLs may represent an early manifestation of PVL, particularly given their high recurrence rates and potential for MT [5, 6, 7, 13].

Several diagnostic criteria for PVL have been proposed over time (e.g., [11, 12, 14, 15]). Although definitions vary in terms and required items, they consistently emphasize clinical progression, multifocality, recurrence, and a tendency toward MT. In our study, we followed a pragmatic approach: cases were classified as PVL when their clinical course fulfilled the items described above, and when histopathology was available, these findings were used to corroborate the clinical impression. The high frequency of multifocal lesions and recurrence observed in our sample is consistent with the features described across these diagnostic criteria. Although MGL has previously been proposed as a distinct PVL subtype, our results suggest that it is better interpreted as a clinical presentation within the PVL spectrum rather than as a separate entity.

The demographic analysis of our patient cohort revealed a balanced distribution between genders and an average age of 60 years, which is consistent with other studies focused on gingival leukoplakia [6, 7, 8]. Interestingly, while tobacco smoking and alcohol consumption are traditionally recognized as risk factors for OL, a significant proportion of our sample did not exhibit these habits [16, 17, 18]. This aligns with prior studies suggesting that PVL and its associated lesions might develop independently of conventional risk factors for oral squamous cell carcinoma [19]. Furthermore, systemic alterations such as hypertension and diabetes were relatively common in our sample.

From a clinical perspective, the predominance of homogeneous lesions in this cohort contrasts with the nonhomogeneous presentation more frequently associated with higher MT risk [18, 20]. However, the presence of PVL in most cases emphasizes that multifocality, rather than homogeneity or nonhomogeneity, may be a stronger indicator of aggressive clinical behavior for these lesions. Additionally, the average lesion size and the involvement of multiple sites suggest that the progression of these leukoplakias is gradual but persistent, underscoring the importance of early diagnosis and intervention.

Histopathological analysis further substantiated the aggressive potential of these lesions. While all examined cases exhibited hyperkeratosis as the most common histopathological finding, agreeing with the study by Alabdulaaly et al. [21], a small subset also displayed varying degrees of epithelial dysplasia, including moderate to severe dysplasia in three cases. These findings reinforce the notion that MGLs, particularly those associated with PVL, might be at a heightened risk of MT, as evidenced by the three cases of gingival squamous cell carcinomas in our study. Another finding observed in 33.3% of the lesions analyzed microscopically was a subepithelial lymphocytic inflammatory infiltrate. This aligns with recent studies suggesting it as a common histopathological feature in early PVL cases [22, 23]. This feature can lead to a misdiagnosis of lichen planus, as reported previously by our group [24].

Despite their clinical significance, there is no established consensus regarding the most effective therapeutic approach for MGLs nor for leukoplakias at other oral mucosa sites. High‐power laser excision has gained popularity in recent years due to its reported benefits, including reduced trans‐ and postoperative complications [25, 26, 27, 28]. Monteiro et al. [29] suggested that laser may be more effective than scalpel excision, while a systematic review by Paglioni et al. [30] found lower recurrence rates with laser treatment compared to traditional methods. Similarly, Arruda et al. [27] supported the use of high‐power diode lasers, noting favorable outcomes for recurrence despite limitations in sample size. These findings collectively underscore the potential of laser technology as a possible preferred therapeutic option for managing gingival leukoplakia.

Our data showed a slightly higher recurrence rate with traditional scalpel excision (80%) compared to diode laser excision (70%). Alabdulaaly et al. [21] suggested that recurrence may result from the difficulty of achieving complete removal of the lesion without extracting the associated teeth, even when employing more radical surgical techniques. This challenge arises from the potential presence of dysplastic cells within the periodontal ligament, which could repopulate the treated surface [21]. In our study, most lesions treated with both modalities were associated with PVL, which is by itself a persistent condition with high recurrence rates, likely accounting for the frequent recurrences observed in our sample [2, 3, 4]. Despite the comparable recurrence with scalpel excision, the benefits of laser excision in reducing patient discomfort and improving healing should not be overlooked, particularly in cases where surgical precision is paramount or in those associated with moderate or severe epithelial dysplasia, which are usually treated with more radical approaches, causing important functional and esthetic damage [29]. It is important to highlight that laser excision may cause thermal damage, potentially altering the histopathological characteristics of the excised tissue [6]. For this reason, it is essential to perform an incisional biopsy and histopathological analysis before selecting laser excision as the treatment method [6].

Within our sample, only three patients had a history of squamous cell carcinoma arising from pre‐existing gingival lesions. While the potential for MT in OL is well established, gingival leukoplakias are less frequently associated with this progression [31]. Recent research into the clinical and histopathological characteristics of OL indicated that the gingiva is among the most prevalent sites for this OPMD, as well as the tongue and buccal mucosa [32, 33]. Nevertheless, the incidence of MT in gingival leukoplakias is significantly lower than that observed in tongue, mouth floor, and buccal mucosa lesions [32, 33]. However, the risk of this outcome should not be underestimated for MGLs, as it is important to note that gingival squamous cell carcinoma tends to exhibit a more aggressive behavior, necessitating radical treatment approaches that may adversely affect the patient's life quality [34]. Its prognosis is influenced by various factors, including the clinical stage at the time of diagnosis, tumor cell differentiation, presence of lymph node involvement, among others [35, 36]. Furthermore, this neoplasm is associated with an increased risk of metastasis due to its anatomical closeness to the periosteum and bone, which allows for easier invasion of these structures by tumor cells [35]. Consequently, vigilant monitoring and appropriate management of these lesions are essential to prevent such adverse outcomes.

The clustering analysis and its heatmap revealed distinct demographic, clinical, and management patterns. Cluster 1, dominated by older women with multifocal, homogeneous lesions and a high prevalence of PVL, strongly aligns with prior evidence linking this lesion subtype to PVL and its aggressive nature [5, 6, 7]. This reinforces the need for careful monitoring of such lesions given their potential for MT. Cluster 2, with balanced representation between male and female patients and a preference for “wait and see,” suggests that this approach may be effective in some cases, especially for non‐PVL homogeneous lesions with no apparent risk factors, such as tobacco smoking. In contrast, Cluster 3, composed largely of male patients with homogeneous leukoplakias and significant tobacco use, reflects findings that this habit might play a role in these lesions' development and progression [16, 17, 18]. Finally, Clusters 4 and 5, primarily consisting of older females, were linked to a higher frequency of laser excision for both homogeneous and non‐homogeneous PVL lesions. These clusters also showed elevated rates of recurrence and MT, highlighting the value of laser treatment as a minimally invasive solution for managing these high‐risk cases [37]. This stratification reflects the heterogeneity of MGLs and highlights the need for tailored treatment approaches.

Despite limitations such as a relatively small sample size, varying follow‐up periods, some of which were too short to observe critical outcomes, and the lack of histopathological analysis for some lesions, our research provides a more robust and critical assessment of this group of leukoplakias. This study's findings suggest that these lesions exhibit clinical and histopathological features which might contribute to a distinct prognosis compared to other leukoplakias. Cluster analysis revealed that MGLs, while affecting a diverse patient group, are strongly associated with classic PVL cases, supporting the hypothesis that they often represent a stage of this condition [5, 6, 7]. Although these lesions may initially resemble benign conditions like frictional hyperkeratosis, they frequently exhibit management challenges, with high recurrence rates. Further studies focused on MGLs are encouraged, for it is important to recognize the need to exercise vigilance in their early identification, accurate diagnosis, and timely management to improve patient outcomes.

## 5 Conclusion

The findings of the current study support previous hypotheses suggesting that MGLs may represent a stage within the PVL spectrum, being strongly associated with patients diagnosed with this condition. Furthermore, histopathological analysis revealed that, although most cases only exhibited features such as hyperkeratosis and epithelial hyperplasia, a small proportion showed varying degrees of epithelial dysplasia, from mild to severe, conferring a risk of MT. Regarding their treatment, laser excision may be the preferred approach for managing MGLs to promote less invasive lesion control, especially in cases associated with epithelial dysplasia. Close monitoring is essential due to their unpredictable clinical behavior.

## Author Contributions

All authors have made substantial contributions to conception and design of the study. D.L.F.F. and C.M.P. have been involved in data collection and data analysis. D.L.F.F., C.M.P., H.K.F.B., A.R.S.S., P.A.V., and M.A.L. have been involved in data interpretation, drafting the manuscript and revising it critically. A.R.S.S., P.A.V., and M.A.L. have given final approval of the version to be published.

## Ethics Statement

This research was carried out following approval from the Research Ethics Committee of the Piracicaba Dental School, University of Campinas (approval number 69909323.5.0000.5418).

## Conflicts of Interest

The authors declare no conflicts of interest.

## Supporting information


**Table S1:** Number of interventions and outcomes of MGLs.

## Data Availability

The data that supports the findings of this study are available in the Supporting Information of this article.
