# Supplementary material for: A Clinicopathological Study of Marginal Gingival Leukoplakia
Source: J Oral Pathol Med. 2025 Sep 4;54(10):1012–20. doi: 10.1111/jop.70052 (PMC12602128; doi:10.1111/jop.70052)
Supplement: Supplementary file 1 — Table S1: Number of interventions and outcomes of MGLs. [file JOP-54-1012-s001.docx]

**Supplementary Table 1.** Number of interventions and outcomes of MGLs

| **Intervention** | **PVL N (%)** | **Non-PVL N (%)** | **Total N** | **Recurrence N (%)** | **Malignant transformation N (%)** |
| --- | --- | --- | --- | --- | --- |
| **Scalpel excision** | 4 (80) | 1 (20) | 5 | 4 (80) | 0 (0) |
| **Laser excision** | 8 (80) | 2 (20) | 10 | 7 (70) | 0 (0) |
| **Wait and see** | 14 (73.7) | 5 (26.3) | 19 | 0 (0) | 1 (5.3) |

Abbreviations: MGL. Marginal gingival leukoplakia; PVL. Proliferative verrucous leukoplakia
